# Vertical, capacitive microelectromechanical switches produced via direct writing of copper wires

**DOI:** 10.1038/micronano.2016.10

**Published:** 2016-04-25

**Authors:** Zhiran Yi, Jianjun Guo, Yining Chen, Haiqing Zhang, Shuai Zhang, Gaojie Xu, Minfeng Yu, Ping Cui

**Affiliations:** 1 Zhejiang Key Laboratory of Additive Manufacturing Materials, Ningbo Institute of Materials Technology and Engineering, Chinese Academy of Sciences, Ningbo 315201, China; 2 D. Guggenheim School of Aerospace Engineering, Georgia Institute of Technology, Atlanta, GA 30332, USA; 3 Nano Science and Technology Institute, University of Science and Technology of China, Suzhou 215123, China

**Keywords:** microelectromechanical switch, direct writing, copper microwire

## Abstract

Three-dimensional (3D) direct writing based on the meniscus-confined electrodeposition of copper metal wires was used in this study to develop vertical capacitive microelectromechanical switches. Vertical microelectromechanical switches reduce the form factor and increase the area density of such devices in integrated circuits. We studied the electromechanical characteristics of such vertical switches by exploring the dependence of switching voltage on various device structures, particularly with regard to the length, wire diameter, and the distance between the two wires. A simple model was found to match the experimental measurements made in this study. We found that the electrodeposited copper microwires exhibit a good elastic modulus close to that of bulk copper. By optimizing the 3D structure of the electrodes, a volatile electromechanical switch with a sub-5 V switching voltage was demonstrated in a vertical microscale switch with a gap distance as small as 100 nm created with a pair of copper wires with diameters of ~1 μm and heights of 25 μm. This study establishes an innovative approach to construct microelectromechanical systems with arbitrary 3D microwire structures for various applications, including the demonstrated volatile and nonvolatile microswitches.

## Introduction

Microelectromechanical systems (MEMS) have been used in many applications in many industries, including the medical, automotive, optics, electronics, and biotechnology sectors^1,2^. MEMS switches operated by radio frequencies are essential components in the circuit architectures of a variety of MEMS-based systems^3^. As an electromechanical switch, MEMS switches offer markedly lower power consumptions, better isolations, and lower insertion losses compared with conventional field-effect transistors or PIN-diode-based switches^3,4^. MEMS switches are typically fabricated with silicon-surface micromachining technology and are typically planar, which requires a large surface budget on integrated circuit chips^5^. Submicron floating cantilevers fabricated with a metal layer (for example, Au, Al, Pt, and so on) are then used as mechanical moving electrodes. However, in thin metal films, it is difficult to control the stress that causes most metal cantilevers to warp upon release^6,7^. To accommodate the further miniaturization and integration of devices and systems, there is a trend towards developing three-dimensional (3D) circuit architectures and related 3D devices, including novel 3D MEMS switches.

Vertical switches that use aligned nanowires and nanotubes have exhibited outstanding capacities in MEMS switches. The simplest microwire-based electromechanical switch consists of two parallel and freestanding microwires that are electrically connected to fixed electrodes. When a voltage is applied between the two microwires, the induced electrostatic force pulls the wires towards each other. The threshold switching voltage is thus defined as that voltage that establishes an ‘on state’ when a higher voltage forms electrical contact between the deflected wires. These wires exhibit high stiffness and large deflection capacities to achieve ultrafast switching response times^8^. In addition, the micro/nano-scale size of these wires is favorable to achieve ultrahigh density and ultralow-energy-consumption switch systems^9^. As a result, capacitive switches that are based on nanowires and nanotubes have been popular for use as robust nanotweezers via electrostatic defection^10,11^. For example, researchers have integrated silicon nanowires^12–16^, silicon carbide (SiC) nanowires^17,18^, and Pt nanowires^19^ into capacitive switches as switching elements. However, most of these studies were based on complex, in-plane micronanolithography technology, which must be performed in a high-vacuum environment with low throughput. As a result, the fabricated parts are restricted to 2D/2.5D microparts, and manufacturing complex 3D microstructure remained a challenge. Recently, Jang *et al.*^8,20^ demonstrated a vertical, three-terminal, carbon-nanotube-based electromechanical switch, in which multiwalled carbon nanotubes were catalytically grown and used as active elements. Similarly, free-standing germanium nanowires^21,22^ and Mo_6_S_3_I_6_ nanowires^23^ were also used as switching elements. Although such microswitches provide many advantages compared with conventional semiconductor switches and are being studied in detail^24–27^, most of those fabrications are incompatible with typical microfabrication processes. To date, precisely controlling nanowire spacing in vertical switching devices remains challenging, and the fabrication process (for example, growing vertically aligned carbon nanotubes^28^ and focused ion beam chemical vapour deposition^29^) remains time consuming and costly. In addition, an undesirablely high actuation voltage is still required to switch the device ON/OFF. Consequently, practical applications of such devices remain limited due to this complex fabrication process. The study of such a simple two-wire switch system should help describe the electromechanical behavior of such a device and other electrical issues related to operating such devices, such as electrical burnout^30,31^.

Metal contact switches have a low on-resistance and a high isolation and are suitable for signals with frequencies including DC, millimeter wave (that is, 100 GHz) and others^32^. It is expected that reducing the gap distance between these wires using 3D structures in switching devices is the most effective means to reduce the threshold switching voltage and thus resolve electrical burnout issues. Among available metals, success in controlling the size and shape of the wires produced has primarily limited the materials used to gold and silver, which has led to a wide range of applications^33–35^. Copper microwires (Cu MWs) are ideal candidate for commercial applications due to their low cost and high abundance compared with those of gold and silver. In addition, Cu exhibits excellent electrical conductivity, malleability, ultrahigh elastic strains and fracture strength^36^. However, synthetic approaches that could precisely control the sizes and shapes of 3D Cu MWs were not well established until recently because of the many challenging issues such as oxidation, dispersity and stability. Thus, the fabrication of 3D Cu microstructures and their incorporation in MEMS switches are critical for the exploitation of future low-cost microdevices.

In this study, we propose the use of a high-efficiency, direct-writing technology that is based on the meniscus-confined electrodeposition principle^37^ to fabricate vertical microelectromechanical switches. We also report on the electrostatic pull-in behavior of 3D-microswitch-based on Cu MWs for the first time. Meniscus-confined electrodeposition has been demonstrated to be an effective direct-writing process for fabricating intricate 3D metal microstructures that do not use complex lithography processes^38^. The vertical Cu MWs were electrodeposited onto surface electrodes using the meniscus-confined electrodeposition method. A glass substrate lithographically patterned with Au/Ni surface electrodes was used as the base for growing vertical Cu MWs. The height and shape of the Cu wires are defined by the designed motion path of the translation stages, and the diameter of the wire is defined by the nozzle size of the glass micropipette used in the system. Cu is used due to its good electrical and mechanical properties.

## Materials and methods

**Figure 1:**
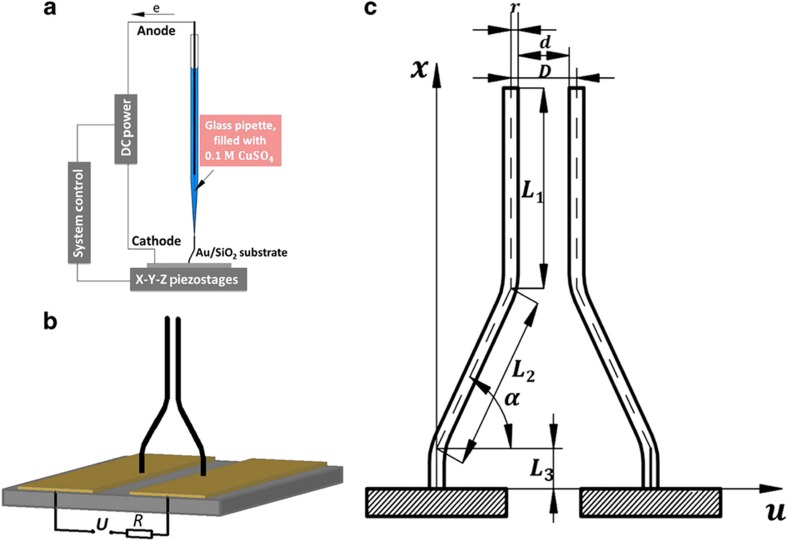
Overview of device fabrication and measurement. (**a**) Schematic of the fabrication process: the Cu MWs were vertically grown from the electrodes using the meniscus-confined 3D electrodeposition approach. (**b**) Structure of NEMS/MEMS devices and the circuit used to characterize the device pull-in voltage. Two gold electrodes were patterned via electron-beam lithography, sputtering and lift off. (**c**) Structural annotation of the device.

Figure 1 shows the proposed 3D design and the fabrication steps required to produce this design. The fabricated device consists of two flexural copper microwires (Cu MWs) that are perpendicular to the substrate surface on the surface electrodes. The microwires were constructed on conducting (gold) surfaces via a meniscus-confined 3D electrodeposition process (that is, a 3D direct-writing process), resulting in the fabrication of complex metallic microarchitectures. It is expected that reducing the gap distance *d* of the vertical aligned microwire to the submicron/nano-scale is required to lower the threshold switching voltage and thus resolve electrical burnout issues in switching devices. This process is challenging and less consistent for vertically aligned 1D microwires. Using intricate 3D wire structures, the proposed design makes the electrodepositional growth of Cu MWs on the substrate more straightforward and convenient to reduce *d* between the microwires. The apparatus shown in Figure 1a consists of an electrolyte-containing micropipette with a microscopic dispensing nozzle, a high-resolution monitoring system, electrical equipment and a central control unit. The glass micropipette, which has an opening diameter of 1 μm, is produced by a pipette puller (P-2000, Sutter Instrument Co., Novato, CA, USA) and is filled with a Cu electrolyte (0.1 M CuSO_4_). The conductive substrate surface (that is, anode) is created at the rear opening of the pipette, and gold-coated glass wafers are used as substrates. The direct-writing process is conducted at a constant relative humidity of 80%. Both the positioning and the pulling speed of the pipette are accurately controlled with a three-axes high-precision nanopositioning piezo stage (P-562.3CD, Physik Instrumente GmbH, Karlsruhe, Germany) with a positioning resolution below 10 nm. An electrical potential between the Au electrode and the substrate was applied using a high-voltage-source measurement unit (237, Keithley, Cleveland, OH, USA). The printing process was observed *in situ* using a high-resolution monitoring system that consisted of an optical objective lens (VMU-V, Mitutoyo, Kawasaki, Japan) and a 3.3-megapixel digital camera (SC30, Olympus, Tokyo, Japan). As shown in Figure 1c, switching devices with different vertical segment lengths *L*_1_, slant segment lengths *L*_2_, slant angles *α* and distances *D* were fabricated. In this study, Cu MWs with defined diameters 2*r* of ~1.0 μm were fabricated and used as active components in the device. The detailed description of the metal microwires growth by this technique can be found elsewhere^38,39^.

The microscopic characteristics of the printed microswitch architectures were analyzed using field emission scanning electron microscopy equipped with an energy dispersive spectrometer (SEM-EDS, FEI QUANTA 250 FEG). The pull-in/pull-out characteristics of the device were investigated by an electromechanical analysis. As shown in Figure 1b, the bottom electrodes are electrically connected to a power supply. A resistor was connected in series with the device to limit the current at the ‘on state’ passing through the device^25^. As the voltage applied across the active element, the resulting electrostatic force is balanced by elastic restoring forces from the deflection of the Cu MWs. When a critical ‘pull-in’ voltage is reached, the electrostatic forces overwhelm the remaining force, and the microwires make electrical contact with each other, establishing the ‘on state’. The switching voltage *V* will be determined by the balance of the electrostatic force between the wires and the elastic force of the wires. The switching voltage was measured by monitoring the sudden rise in the current passing through the device. All electrical measurements were performed at room temperature and pressure on a probe station with a flow of dry nitrogen flooding the inner chamber. The size dependencies of the pull-in voltage were predicted by the finite element method.

## Results and discussion

### Manufacturing 3D Cu microswitches on device substrates

**Figure 2:**
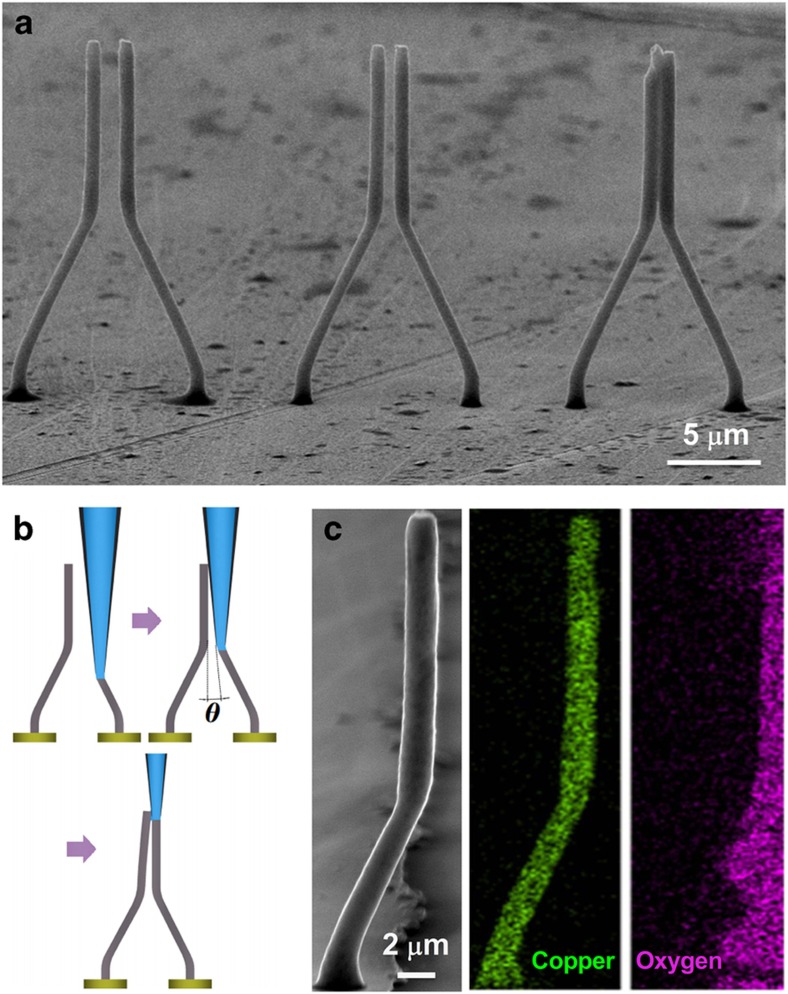
Growth evolutions of 3D microswitches. (**a**) Micrographs of switches with different active gap distances *d*, ranging from 2 to 0.5 μm (left to right). (**b**) Schematics of the failure mode due to liquid-bridge formation during the fabrication process; (**c**) Energy dispersive spectrometer (EDS) maps of a single microwire.

To demonstrate the proposed technique, the effectiveness of the meniscus-confined electrodeposition for Cu-based microswitches were studied and evaluated. In the proposed process, Cu MWs with diameters of ~1 μm and heights of 20 μm are grown using a micropipette with a diameter of ~1 μm filled with 0.1 M CuSO_4_ aqueous solution. Because metal growth is confined within the meniscus, which can be between several microns and 100 nm in size, the number of nucleation sites and the size of the grains are controlled by small-scale electroactive areas during growth. We have confirmed that the nanowire has a nanocrystalline structure with an average grain size of tens nanometres^39^. The nanocrystalline structure results in a smooth surface^37,39^, and ultrahigh elastic strains and fracture strengths were reported to be sustained in Cu nanowires due to grain boundary strengthening^40,41^. This feature can be beneficial for the operating reliability of MEMS switches. Three-dimensional (3D) metallic microarchitectures with diverse and complex features can be printed by accurate handling of the electrolyte meniscus during the printing process. As shown in Figure 2, complex 3D electrodes with high aspect ratios and truly 3D microstructures were built at desired locations by moving the electrode with respect to the substrate. The different lengths and angles used depended on the different moving path applied, which can be realized using an automated computer-controlled system. The electrodes were found to stick to each other as the electrode distance decreased to ~0.5 μm. As shown in Figure 2b, steric constraints between the glass micropipette and the adjacent electrode will occur as the active gap *d* decreases due to the shape of the glass micropipette used. When *d* decreased to the threshold distance, the liquid bridge between the dispensing nozzle and the adjacent electrode becomes more prevalent due to capillary forces, causing irreversible adhesion to occur (for example, the rightmost pair Cu MWs shown in Figure 1a). Thus, the gap distance should satisfy *d*>*L*_1_ tan *θ*, where *θ* is the slant angle of the pipette wall shown in Figure 2b. In the experiments in this study, *θ* is ~3° for the 1-μm-diameter micropipette, and the gap distance should be more than approximately 520 nm to avoid adhesion. It has been reported that reducing the gap distance can effectively decrease the pull-in voltage and mitigate damage in switching devices^9,17,42,43^. As a result, it is impossible to further decrease the active distance. As discussed in the following section, we can decrease the switch-on voltage to several volts by optimizing the 3D electrode structures.

The propensity of Cu to oxidize under ambient conditions is an imminent challenge for the applicability of Cu nanostructures. To prevent surface oxidation, the synthesis of Cu nanostructures must be performed under inert atmosphere and reducing environments. However, the energy dispersive spectrometer (EDS) maps of copper and oxygen elements in a single microwire, as shown in Figure 2c, provide evidence for the marked oxidation resistance of the proposed process, despite fabrication occurring in ambient conditions. Thus, Cu MWs were produced via the 3D direct-writing process. In general, devices at this scale are sufficient for reliable operation and special needs; structural optimization is used, as shown in the following section.

### Nonvolatile switching behavior

**Figure 3:**
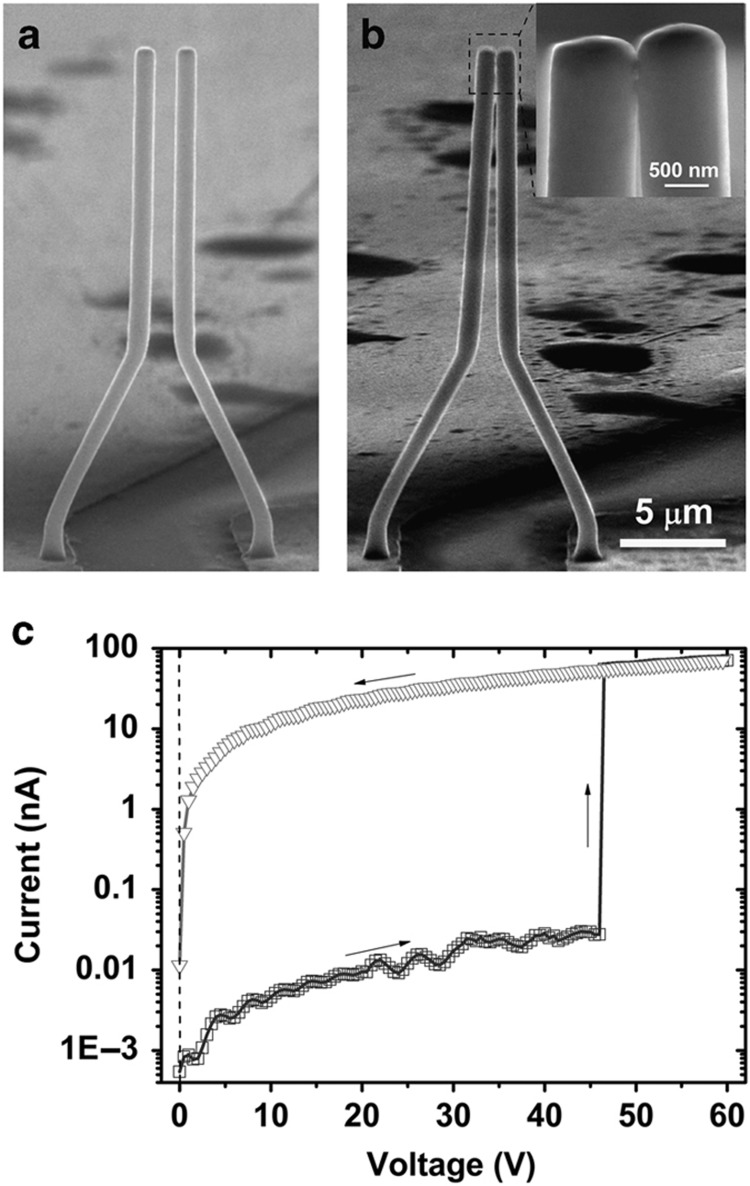
MEMS switch based on two symmetrical Cu MWs. (**a** and **b**) SEM image of the device before supply and after the removal of a bias voltage. The scale bar corresponds a length of 5 μm. (**c**) Plot of current versus voltage for the device exhibiting nonvolatile behavior. Sizes of the device are described by the following parameters: *α*=63°, *L*_1_=15 μm, *L*_2_=8.944 μm, *L*_3_=2 μm (always), and diameter=950±5 nm.

Figure 3a shows the SEM image of a typical switch device operated at a high switching voltage due to the initially large gap distance. The *I*−*V* curve shown in Figure 3c shows a nonvolatile switching behavior with a threshold voltage of 46.5 V, which is higher than other reported Ge-based devices^21^ and carbon-nanotube-based electromechanical switches^20^. Upon switching to the ‘on state’, an Ohmic contact between the two Cu wires was observed with an overall contact resistance measured to be ~2.0 MΩ. The microwires did not return to their original position and remained stuck together, even after the bias voltage was removed. It is known that a high bias voltage can induce damage to the switching device^9,26^. The high-magnification SEM image shown in Figure 3b indeed shows two joining bridges in the contact area between the wires, indicating a burnout, which resulted in effectively spot welding the two wires together. The van der Waals attractive force between the wires was found to not be responsible for the bonding of the two wires both from a simple quantitative estimation and experimentally. In the experiment, one of the Cu wires was pushed into contact with the other wire using a manipulator; no clear bonding was observed. This feature of maintaining a permanent electrical connection with the application of a set bias could be useful in microdevices that require nonvolatile switching.

### Tuning the design parameters of the nonvolatile switches

To optimize the design parameter of the switches, we studied the electromechanical response of the Cu microwire switch experimentally and analytically. To investigate the instability of the switches, the numerical finite element method was used. The total potential energy of the system consists of the contributions from the mechanical energy^44–46^ due to the deflection of the wire and the electrostatic energy^10,45^ due to the capacitive interaction between the wires. This energy can be described as follows:

(1)E(u)=πEr44∫0xtip(d2udx2)2dx−12C(u)V2

where *E* is the Young’s modulus of Cu, *r* is the radius of the wire, *x* is the vertical coordinate of the microwire, *u* is the deflection of the microwire from the original position, *V* is the applied static voltage between the microwires, *C* is the capacitance between the two Cu MWs, which is defined as follows^47,48^:

(2)C=∫0xtipπεrε0arcosh(D'−2u2r)dx

$$\;D\prime =\{\begin{array}{l} D+2L_{2}\;\cos\alpha ,\;x\in [0,L_{3}]\\ D+2(L_{2}\;\cos\alpha -\frac{x-L_{3}}{\tan\alpha }),\;x\in (L_{3},\;L_{3}+L_{2}\;\sin\;\alpha )\\ D,\;x\in [L_{3}+L_{2}\sin\alpha ,L_{3}+L_{2}\;\sin\alpha +L_{1}] \end{array},$$

$$\;L_{3}=\text{2}\;µ\text{m}$$

where *ε*_*r*_ and *ε*_0_ are the relative permittivity of air (*ε*_*r*_=1) and the permittivity of vacuum (*ε*_0_=8.854×10^−12^ F m^−1^), respectively. Using the minimum energy principle, *δE*(*u*)=0, the following equation can be obtained:

(3)d4udx4=8εrε0V2Er4(D'−2u)2−4r2⋅acosh2(D'−2u2r)

The dimensionless form of the governing equation of Cu MWs is shown above, and the boundary value problem for each microwire can be defined as follows:

(4){u(0)=dudx(0)=0d2udx2(xtip)=d3udx3(xtip)=0

**Figure 4:**
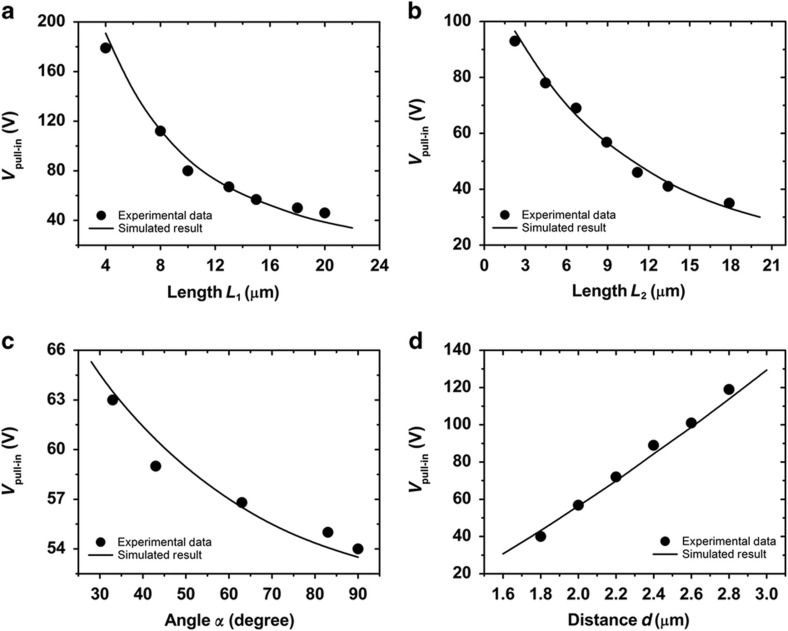
Effects of length (**a** and **b**), angle *α* (**c**) and distance of the vertical segment (**d**) on the pull-in voltages of the microwires switches. Except for those indicated in the horizontal coordinate, the sizes of the device are fixed (that is, *α*=63°, *L*_1_=15 μm, *L*_2_=8.944 μm and *d*=1.0±0.02 μm).

Figure 4 shows the modeled result and its comparison with experimental measurements. These devices were fabricated, and their dimensions were calibrated in a scanning electron microscope. Their switching behaviors were then measured in a clean ambient environment. Figures 4a and b depict the dependence of the pull-in voltage of the device on the segment length of the microwires. As expected, the required pull-in voltage decreases as the segment lengths increase due to the reduced elastic stiffness of the microwires. The pull-in voltage is shown to be more sensitive to changes in the segment length *L*_1_ due to the narrower gap distance between them, thus creating a larger electrostatic force. Figure 4c shows the effects of the angle *α* on the pull-in voltages of the device. As the angle *α* increases, the horizontal bending stiffness of the *L*_2_ segment decreases in the *u* direction, and consequently, the required pull-in voltage slowly decreases. The primary reason for this phenomenon is that the moment of inertia decreased due to a change in the horizontal cross-section of the vertical wires^49^. The pull-in voltage is shown to be most sensitive to the distance between the *L*_1_ segments, as shown in Figure 4d, because the capacitance between the microwires exhibits a virtually inverse dependence on the gap distance and thus the electrostatic force upon the applied voltage. Electrostatically actuated test structures can describe the MEMS system’s material property^43^. In all cases, the modeling results agreed with the experimental results, indicating both the good mechanical and electrical characteristics of the fabricated microstructure.

**Figure 5:**
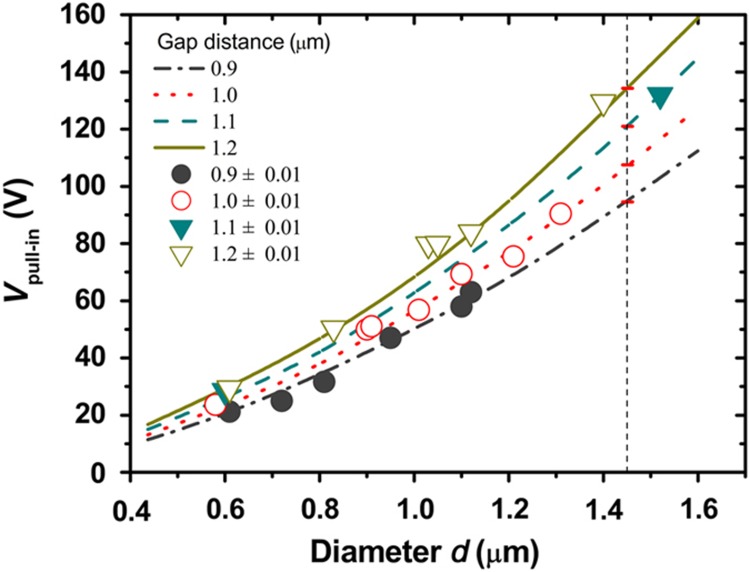
Effects of the Cu MWs’ diameter on the pull-in voltages of the microwire switches. Other sizes of the device are fixed (*α*=63°, *L*_1_=15 μm and *L*_2_=8.944 μm). The lines represent the simulated results, and the dots represent the experimental data.

To describe the effect of the Cu MWs’ diameters on the pull-in voltage in detail, a total of 22 devices with different diameters were investigated; the detailed pull-in voltages of those devices are shown in Figure 5. In this study, the diameters of Cu MWs varied from ~0.4 to 1.6 μm, and the response of the pull-in voltage was approximately 10 V per 100 nm. It was found that the pull-in voltages of the devices increase as their diameters increase. This change in the pull-in voltage with the diameter is relatively slow when the diameters of the Cu MWs are small. From Equation (3), the pull-in voltage approximately follows $V\propto r^{5/2}$ as the gap distance *d* approaches the diameter 2*r*. Concurrently, the gap distance exhibits an approximately linear change in the pull-in voltage when the diameter is constant, which is also shown in Figure 5. This result agrees with the description shown in Figure 4d. The suitable diameter of Cu MWs strongly affects high-performance devices; this primarily depends on the mechanical strength of the Cu MWs used, which is depends on their diameters^50^. We measured the Young’s modulus of the electrodeposited Cu microwires using the well-known electric-field-induced resonance method^51–54^. The measured Young’s modulus of an individual Cu MW that is 100 μm in length and 0.85 μm in diameter is shown to be 122.6 GPa (see Supplementary Information), which is near the simulated results of 119.8 GPa for Cu wires with diameters ranging from 0.4 to 1.6 μm, as shown in Figure 5. It was unexpectedly found that the mechanical properties of the electrically deposited Cu wires closely approximate those of the bulk Cu. The reported elastic modulus and Young’s modulus of Cu nanowires were between 160 and 105 GPa as the diameter increased from 100 to 350 nm^55^. The mechanical properties of the Cu MWs were found to approach those of bulk Cu when the radius exceeded 350 nm^56^. In the proposed approach, meniscus-confined electrodeposition typically produced a confined nanocrystalline wire structure. As a result, the mechanical properties of the material are reasonably assumed to be near to those of bulk polycrystalline Cu because many material properties are strongly dependent on grain size, such as strength, ductility, hardness, and electrical resistivity^57–59^.

### Volatile switching behavior of optimized switches

**Figure 6:**
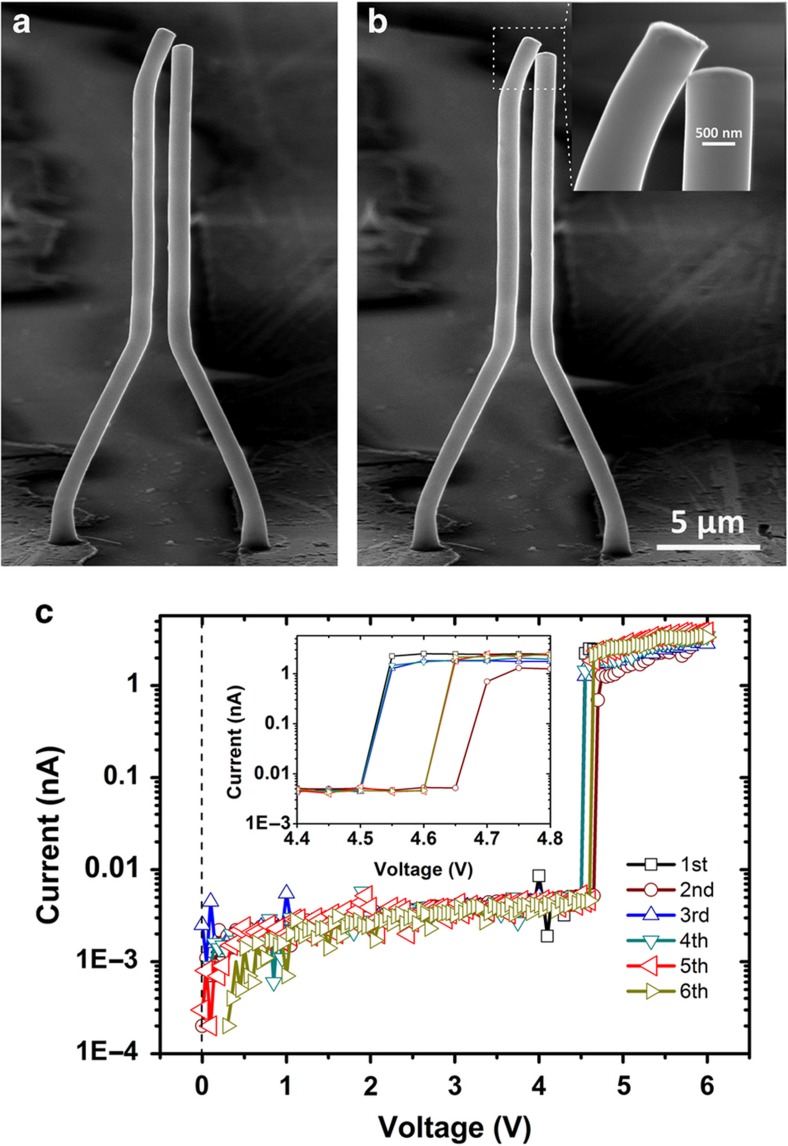
MEMS switch with an additional slant segment. (**a** and **b**) Scanning electron micrographs of the device before and after the supply of a bias voltage, respectively. The *α*, *D*, *L*_1_, *L*_2_, and diameter of device are equal to 63°, 2 μm, 15 μm, 8.944 μm, and 1.02±0.005 μm, respectively. (**c**) *I*–*V* characteristics of the device in multiple operations. The scale bar corresponds to a length of 5 μm.

The highly sensitive dependence of the pull-in voltage on the gap distance can be further exploited to produce a vertical volatile switch with a sub-5 V switching voltage, which may ultimately be useful even in high-density, integrated logic devices. We took advantage of the direct-writing capability of the proposed fabrication technique to fabricate such a 3D vertical switch with a gap distance at the nanometre scale. Figure 6 shows such a vertical switch with a gap distance of ~100 nm that was created via the direct-writing method with an additional slant segment to reduce the distance between the tips of the microwire pair. This vertical switch was found to establish an ‘on state’ at a relatively small bias voltage of ~4.5 V in the first run, as shown in Figure 6c. After removing the applied bias voltage, the microwires deflected immediately back to their original position, which was indicative classic volatile switching behavior. A relatively large contact resistance of ~650 MΩ was found between the microwires that made electric contact. The existence of such a large contact resistance may also be responsible for maintaining a finite voltage drop across the contact and thus a potential difference between the Cu MWs. Therefore, even with electric contact between the microwires, a finite electrostatic force between the microwires, albeit marginally reduced from that before making the electric contact, still remained. This may explain why the microwires deflected back to their original position only when the bias voltage is fully removed instead of deflecting back at the instant of making electric contact, as in typical switching devices with a low contact resistance. In the following switching operations, the pull-in voltage was found to fluctuate due to certain nanoscopic structural changes near the contact site; the amplitude of the change in the switching voltage was found to be ~0.15 V, as shown in Figure 6c. The volatile switching behavior of this vertical switch can be further improved by first reducing the contact resistance between the microwires by coating metals with low contact resistances, such as Au, Pt, Ni or Co, and by further optimizing the mechanical and structural design of the vertical switch.

## Conclusion

Novel MEMS switches with true 3D copper-wire-type electrodes were fabricated using the meniscus-confined 3D electrodeposition method. The performance of these devices was found to depend on the dimensional structure of the electrodes, including the active length, the slant angle, the diameter and the distance between the two Cu MWs. The theoretical analysis indicated that the Young’s modulus of Cu MWs was ~120 GPa. Therefore, Cu MWs with diameters between 0.4 and 1.6 μm were produced via 3D direct writing and exhibited elastic moduli near that of bulk Cu. By optimizing the electrode structure, both volatile and nonvolatile switching behaviors were obtained. A volatile MEMS switch with a sub-5 V switching voltage was also demonstrated. The low actuation voltage of the proposed MEMS switch can be used in more novel microdevices designs and applications. Further study should investigate the response behavior and practical application of such vertical switches with low switching voltages that are fabricated using this direct-writing method.
