# Supplementary material for: Vertical, capacitive microelectromechanical switches produced via direct writing of copper wires
Source: Microsyst Nanoeng. 2016 Apr 25;2:16010. doi: 10.1038/micronano.2016.10 (PMC6444713; doi:10.1038/micronano.2016.10)
Supplement: Supplementary Information [file micronano201610-s1.pdf]

Supplementary file

Vertical, capacitive microelectromechanical switches produced via direct writing of copper wires

Zhiran Yi[1,3], Jianjun Guo[1], Yining Chen[2], Haiqing Zhang[1], Shuai Zhang[1,3], Gaojie Xu[1], Minfeng Yu[2] and Ping Cui[1]

Microsystems & Nanoengineering (2016) **2,** 16010; doi:10.1038/micronano.2016.10

MEASUREMENT OF THE MECHANICAL PROPERTIES

The well-known electric-field-induced resonance method[1–4] was mainly employed in our experiments for measuring the Young's modulus of copper microwires. The Young's modulus of an individual microwire can be obtained

$$E=\left(\frac{8\pi L^2\upsilon}{\beta^2 D}\right)^2\rho \qquad (S1)$$

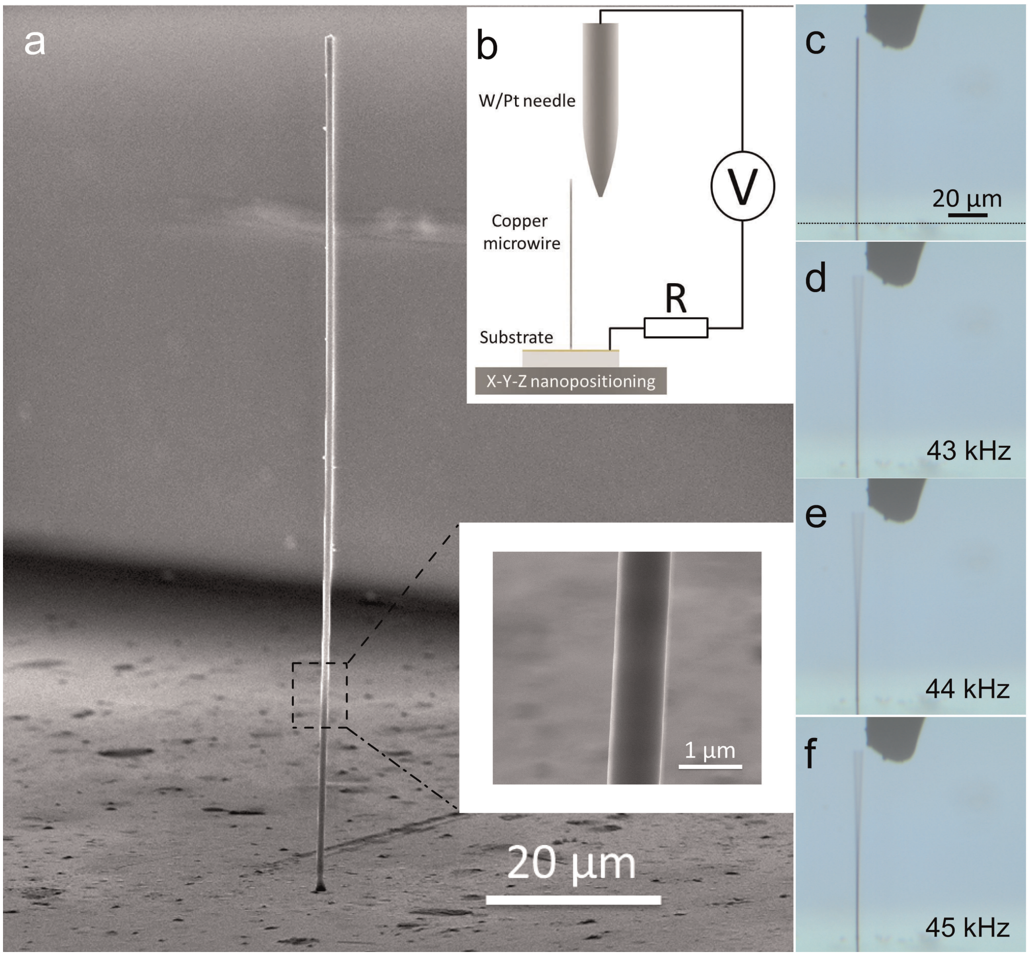

Figure S1 Copper microwire response to resonant alternating applied potentials. (**a**) SEM image showing copper microwire (diameter of this microwire is 850 ± 10 nm). (**b**) Schematic diagram of the measurement of the mechanical properties of the single microwire. (**c**) The optical microscopy image for the microwire and the needle tip. (**d**-**f**) Resonant excitation (the natural resonance frequency of the beam $\upsilon \approx 44$ kHz).

[1]Zhejiang Key Laboratory of Additive Manufacturing Materials, Ningbo Institute of Materials Technology and Engineering, Chinese Academy of Sciences, Ningbo 315201, China; [2]D. Guggenheim School of Aerospace Engineering, Georgia Institute of Technology, Atlanta, Georgia 30332, USA and [3]Nano Science and Technology Institute, University of Science and Technology of China, Suzhou 215123, China
Correspondence: Jianjun Guo (jjguo@nimte.ac.cn) or Minfeng Yu (minfeng.yu@ae.gatech.edu)

where constant β is 1.875 for the first harmonic mode. L, D, and ρ are the length, the diameter, and the mass density of the beam respectively. υ is the natural resonance frequency of the beam. It is notable that the accurate measurements of beam size and the natural frequency are essential for reaching the exact Young's modulus. Figure S1 shows a single microwire with 100 μm length, 0.85 μm diameter and the natural resonance frequency approximates to 44 kHz. Therefore, the Young's modulus of the copper microwire is 122.6 GPa near bulk copper from the equation (S1). The bias voltage was $V = V_s + V_d \sin(2\pi\upsilon t)$, V_s is a constant voltage (50 V) and V_d is a small excitation voltage (5 V).

REFERENCES

1 Poncharal P, Wang ZL, Ugarte D *et al.* Electrostatic deflections and electromechanical resonances of carbon nanotubes. *Science* 1999; **283**: 1513–1516.
2 Suryavanshi AP, Yu MF, Wen JG *et al.* Elastic modulus and resonance behavior of boron nitride nanotubes. *Applied Physics Letters* 2004; **84**: 2527–2529.
3 Ni H, Li XD, Cheng GS *et al.* Elastic modulus of single-crystal GaN nanowires. *Journal of Materials Research* 2006; **21**: 2882–2887.
4 Henry T, Kim K, Ren Z *et al.* Directed growth of horizontally aligned gallium nitride nanowires for nanoelectromechanical resonator Arrays. *Nano Letters* 2007; **7**: 3315–3319.

doi:10.1038/micronano.2016.10
